# Effect of Transmission Reduction by Insecticide-Treated Bednets (ITNs) on Antimalarial Drug Resistance in Western Kenya

**DOI:** 10.1371/journal.pone.0026746

**Published:** 2011-11-11

**Authors:** Monica Shah, Simon Kariuki, Jodi Vanden Eng, Anna J. Blackstock, Kimberly Garner, Wangeci Gatei, John E. Gimnig, Kim Lindblade, Dianne Terlouw, Feiko ter Kuile, William A. Hawley, Penelope Phillips-Howard, Bernard Nahlen, Edward Walker, Mary J. Hamel, Laurence Slutsker, Ya Ping Shi

**Affiliations:** 1 Division of Parasitic Diseases and Malaria, Center for Global Health, Centers for Disease Control and Prevention, Atlanta, Georgia, United States of America; 2 Atlanta Research and Education Foundation, Atlanta, Georgia, United States of America; 3 Center for Global Health Research, Kenya Medical Research Institute, Kisumu, Kenya; 4 Malaria Epidemiology Unit, Liverpool School of Tropical Medicine, Liverpool, United Kingdom; 5 Child Survival and Development Cluster, UNICEF, Jakarta, Indonesia; 6 Centre for Public Health, Liverpool John Moores University, Liverpool, United Kingdom; 7 President's Malaria Initiative, Washington, D. C., United States of America; 8 Department of Microbiology and Molecular Genetics, Michigan State University, East Lansing, Michigan, United States of America; Université Pierre et Marie Curie, France

## Abstract

Despite the clear public health benefit of insecticide-treated bednets (ITNs), the impact of malaria transmission-reduction by vector control on the spread of drug resistance is not well understood. In the present study, the effect of sustained transmission reduction by ITNs on the prevalence of *Plasmodium falciparum* gene mutations associated with resistance to the antimalarial drugs sulfadoxine-pyrimethamine (SP) and chloroquine (CQ) in children under the age of five years was investigated during an ITN trial in Asembo area, western Kenya. During the ITN trial, the national first line antimalarial treatment changed from CQ to SP. Smear-positive samples collected from cross sectional surveys prior to ITN introduction (baseline, n = 250) and five years post-ITN intervention (year 5 survey, n = 242) were genotyped for single nucleotide polymorphisms (SNPs) at *dhfr-*51, 59, 108, 164 and *dhps*-437, 540 (SP resistance), and *pfcrt-*76 and *pfmdr1*-86 (CQ resistance). The association between the drug resistance mutations and epidemiological variables was evaluated. There were significant increases in the prevalence of SP *dhps* mutations and the *dhfr/dhps* quintuple mutant, and a significant reduction in the proportion of mixed infections detected at *dhfr*-51, 59 and *dhps*-437, 540 SNPs from baseline to the year 5 survey. There was no change in the high prevalence of *pfcrt-*76 and *pfmdr1*-86 mutations. Multivariable regression analysis further showed that current antifolate use and year of survey were significantly associated with more SP drug resistance mutations. These results suggest that increased antifolate drug use due to drug policy change likely led to the high prevalence of SP mutations 5 years post-ITN intervention and reduced transmission had no apparent effect on the existing high prevalence of CQ mutations. There is no evidence from the current study that sustained transmission reduction by ITNs reduces the prevalence of genes associated with malaria drug resistance.

## Introduction

Worldwide, three billion people are at risk of malaria infection. Over 85% of the approximately 250 million cases and one million deaths due to malaria occur in Africa each year [1]. Over the past decade, the international community has emphasized the use of malaria prevention to reduce the global burden of malaria as well as to preserve the efficacy of the limited set of antimalarial treatment drugs that are threatened by the emergence and spread of drug resistance [2], [3]. Current World Health Organization (WHO)-recommended malaria control strategies include prompt access to effective treatment, vector control with long lasting insecticide-treated bednets (LLITNs) and indoor residual spraying (IRS), and prevention of malaria in pregnancy [1]. ITNs, in particular, are powerful and cost-effective malaria control tools [4], [5]. In sub-Saharan Africa, the use of ITNs has been associated with a 70–90% decrease in malaria transmission, reduction in childhood malaria morbidity and all-cause mortality, and significant decrease in adverse effects of malaria in pregnancy [6], [7], [8].

Despite the clear benefit of ITNs on the prevention and control of malaria, the impact of transmission reduction by ITN use on the prevalence of antimalarial drug resistance genes in the areas where parasite resistance has emerged due to drug pressure is not well-known. To date, only a limited number of studies have examined the effect of vector control interventions on the spread of antimalarial drug resistance and these studies have yielded conflicting results. A study conducted in Tanzania found that short-term use of ITNs was associated with decreased prevalence of the dihydrofolate reductase (*dhfr*) triple mutant, which is associated with resistance to the antimalarial drug sulfadoxine-pyrimethamine (SP) [9]. After an IRS campaign conducted in Zimbabwe, participants in sprayed villages had a lower risk of chloroquine (CQ) treatment failure and lower prevalence of gene mutations in the parasites conferring resistance to CQ compared to unsprayed villages [10]. However, a study examining the effect of long-term use of insecticide-treated curtains (ITCs) on antimalarial drug resistance in Burkina Faso revealed no changes in the risk of CQ treatment failure and prevalence of gene mutations linked to CQ and SP in intervention compared to control villages [11]. Collectively, these results suggest that decreased transmission by vector control may reduce the number of people exposed to drug resistant parasites and the use of antimalarial treatment, and thus lower drug pressure [9], [10], [11]. However, parasite and human host factors , the genetic basis for drug resistance, and local human migration might also affect the relationship between transmission intensity and drug resistance [12].

Recent theoretical models [3], [13] based on several field studies describe the role of transmission intensity on the spread of drug resistance as indirect. The intensity of transmission affects three main epidemiological mediators: multiplicity of parasite clones, infection risk, and acquired immunity. These three mediators could respectively modulate the degree of sexual recombination of parasites and the intrahost competition between co-infecting parasite clones (intrahost dynamics), the level of drug use in the population, the proportion of malaria infections treated, and the number of parasites in a human host [3], [12], [13]. Additionally, the relationship between transmission intensity and the spread of drug resistance depends on whether resistance to a drug is encoded by a single gene or by multiple genes. Some field evidence and theoretical models have suggested that in the presence of intrahost dynamics the rate of drug resistance, if the genetic basis of resistance is monogenic, increases linearly from low to high transmission intensity. However, drug resistance encoded by two or more genes evolves faster in areas at the extremes of transmission intensity, low and high, compared to intermediate transmission [3], [13], [14].

At the molecular level, drug resistance develops through a series of sequential mutations in genes that synergistically confer resistance. Point mutations in *Plasmodium falciparum dhfr* and dihydropteroate synthase (*dhps*) genes correlate with resistance to pyrimethamine and sulfadoxine, respectively, while mutations in chloroquine related transporter (*pfcrt*) and multidrug resistance (*pfmdr1*) genes are linked with resistance to CQ [15]. In Africa, the *dhfr* triple mutant, Asn-108/Ile-51/Arg-59, has been strongly associated with clinical resistance to SP and the addition of the *dhps* double mutant, Gly-437/Glu-540, creates the quintuple mutant that is associated with *in vivo* SP treatment failure [16]. Mutations at Tyr-86 in *pfmdr1* and at Thr-76 in *pfcrt* genes are associated with CQ resistance [17].

A better understanding of the relationship between the intensity of transmission and the spread of genes that confer resistance to antimalarial drugs could have significant public health implications, as the impact of transmission-reducing interventions may award additional benefits if reduced transmission decreases the spread of drug resistance. However, the relationship between transmission intensity and drug resistance is complex and depends on a number of factors; some can be measured directly while others can only be estimated through proxies [12]. In the present study, the impact of sustained transmission reduction by ITNs and first line drug policy change from CQ to SP on the prevalence of *P. falciparum* gene mutations associated with drug resistance to SP and CQ was investigated during a large bednet trial conducted in children under the age of five between 1996 and 2001 in western Kenya. Using genetic, clinical, and epidemiological data, this study (1) determined the prevalence of gene mutations associated with SP and CQ drug resistance before and five years after ITN intervention and (2) examined the association between epidemiological variables and mutations in the SP and CQ-linked drug resistance genes.

## Materials and Methods

### Study Site and Population

This study was part of a two-phase ITN trial carried out by the Kenya Medical Research Institute (KEMRI) and US Centers for Disease Control and Prevention (CDC) between 1996 and 2001 in Asembo area, western Kenya, where malaria is holoendemic. Detailed methods for the ITN trial are described elsewhere [18], [19]. During the trial, biannual population censuses and annual cross-sectional surveys were conducted in 60 villages between March and May (rainy season) to determine the impact of ITNs on malaria-related morbidity and all-cause mortality in children under the age of five. At each cross-sectional survey, blood samples were collected, and parasitological, clinical, demographic, and entomological information were recorded. *P. falciparum* accounted for approximately 98% of malaria infections in the trial area [18]. For this study, 259 *P. falciparum* smear-positive blood samples collected just prior to ITN introduction as baseline (year 1996 in which the original survey only enrolled children under the age of three years) [20] and 244 samples collected five years post-intervention as ‘year 5 survey’ (year 2001 in which the original survey recruited children under the age of five years) [18] were randomly selected from the same subset of villages. The entomologic inoculation rate (EIR) was recorded at 61.3 and 1.3 bites per person per year at baseline and five years after ITN introduction, respectively [18], [21]. Parasite prevalence in children under the age of five decreased from 70% prior to the trial to 34% at the year 5 survey [18], [22]. ITN usage in children younger than five years of age increased from <5% at baseline to 82.5% at the year 5 survey [18]. Overall, the number of people seeking antimalarial treatment after ITN introduction decreased [22], [23]. During the ITN trial, the national first line treatment for uncomplicated malaria in children changed from CQ to SP in 1998. Prior to the policy change and implementation of ITNs (year 1996), SP was sporadically available in health facilities and prescribed occasionally (<1%) in the study area [23]. In addition, cotrimoxazole (CTX, trimethoprim-sulfamethoxazole), a common antifolate antimicrobial, acts as an antimalarial by inhibiting the same enzymes in the folic acid biosynthetic pathway as SP. The change from penicillin to CTX for first line treatment of respiratory illnesses occurred in the mid-late 1990s in Kenya.

This study was approved by the Ethical Review Committee of the KEMRI, Nairobi, Kenya, the Institutional Review Boards of Michigan State University, East Lansing, MI, and the Centers for Disease Control (CDC) Atlanta, Georgia. Written informed consent was obtained from legal guardians of each child that participated in this study.

### Laboratory Procedures

#### DNA extraction

DNA was purified from red blood cell pellets using the QIAamp DNA Blood Mini Kit (Qiagen, CA, USA).

#### SNP genotyping of drug resistance markers

Real-time polymerase chain reaction (PCR) (Stratagene Mx3005P, CA, USA) was used to detect single nucleotide polymorphisms (SNPs) at *dhfr*-51, 59, 108 and 164, *dhps*-437 and 540, *pfcrt*-76 and *pfmdr1*-86 using published procedures [24], [25], [26]. Briefly, standards and field samples were run in duplicate in 25 µL reactions containing TaqMan Universal Mastermix (Applied Biosystems, CA, USA), 2 µL of DNA (diluted 1∶10), gene-specific forward and reverse primers, and SNP-specific TaqMan MGB probes (Applied Biosystems, CA, USA). Four ten-fold serial dilutions of both wild type and mutant parasite laboratory strain standards, depending on the SNP, and negative control templates were run on every plate as positive and negative controls.

### Definitions

#### Genetic definitions

For all eight SNPs genotyped, samples were classified as (1) pure or mixed and (2) wild type or mutant. A mixed sample contained PCR amplification of both wild type and mutant strains with the minor strain >30% of the major strain [27]. SP genotypes (*dhfr*, *dhps*, and combined *dhfr/dhps*) were determined according to the criteria outlined by Kublin and colleagues [16]. Briefly, *dhfr* genotype, based on mutations in *dhfr*-51, 59, and 108, was classified as wild type, single, double, and triple (pooled triple mixed and pure), *dhps* genotype (mutations in *dhps*-437 and 540) as wild type, single, and double (pooled double mixed and pure), and combined *dhfr*/*dhps* genotype (mutations in *dhfr*-51,59,108 + *dhps*-437,540) as wild type, single, double, triple, quadruple, quintuple (pooled quintuple mixed and pure). For SNPs linked to CQ resistance, *pfmdr1-*86 and *pfcrt-*76 were analyzed separately and genotypes were defined as wild type or mutant. Furthermore, CQ and SP gene combined haplotypes were defined based on SNP results for samples that were single infection by RT-PCR at *pfcrt-*76, *pfmdr1-*86, *dhfr-*51, 59,108, and *dhps-*437,540 [28], [29].

As a previous study from our group using a subset of samples showed that there was no change in multiplicity of infection (MOI) measured by neutral microsatellites (MS) between baseline and the year 5 survey [30], MOI data using the neutral MS markers were not collected for the current study. Therefore, we estimated the prevalence of mutations [16], which reflects the proportion of human blood samples containing the mutation, rather than the frequency of mutations, which measures the proportion of malaria clones carrying a mutation [27]. Mixed infections for all SNPs were considered mutant infections when calculating the prevalence of SNP mutations. The proportion of mixed infections was defined as the number of mixed infections divided by the total number of mutations (pure and mixed) in each SNP.

#### Clinical definitions

Epidemiological variables that were of interest in the analysis included age, sex, parasite density, hemoglobin level (g/dL), presence of gametocytes, report of fever in previous 48 hours, geographic information system (GIS) distance (in meters) to the Lake Victoria shore, nearest clinic, nearest compound and elevation, and SP, CQ, and CTX drug use within two weeks prior to surveys. As CTX has anti-malarial properties through the same mechanism as SP, we also combined the usage of SP and CTX as a single variable which we refer to as antifolate to assess the relationship between antifolate drug pressure and SP gene mutations.

### Data Analysis

Differences between participant characteristics at baseline and five years post-intervention were analyzed using chi-square and two-sample t-tests (Satterthwaite's statistic). Parasite density and Euclidean distance to nearest compound were log transformed prior to statistical testing. Differences in the prevalence of SNP mutations, proportion of mixed infections in total mutations, and prevalence of SP genotypes between baseline and post-intervention samples were examined using chi-square tests. No adjustments for multiple comparisons were made, as tests for each molecular marker were considered independent.

To explore the association between epidemiological variables and drug resistance genotype, univariable and multivariable binary or cumulative logistic regression were used. As the mutations in *dhfr* and *dhps* that confer resistance to SP occur stepwise and sequentially and many studies have shown that higher levels of resistance to SP occur when more mutations are introduced, the outcome was modeled as an ordinal response variable using cumulative logistic regression when other model assumptions were met. *Dhfr* genotype was analyzed using logistic regression by collapsing into two categories in order to ensure sufficient sample size in each category for analysis: (1) wild type, single, and double collapsed, and (2) triple mutations. *Dhps* genotype was analyzed as three categories in the cumulative logistic model: (1) wild type, (2) single, and (3) double mutations. *Dhfr* and *dhps* combined genotype was classified into three categories to ensure adequate sample size in each group and analyzed using the cumulative logistic regression: (1) wild type, single, double, and triple collapsed, (2) quadruple, and (3) quintuple mutations. Two different multivariable models were used, one with antifolate use (SP and/or CTX) and another with only SP use as explanatory variables for drug use, due to expected multicollinearity if both variables were added to the same model. For each cumulative logistic model, the proportional odds assumption was evaluated using the score test, where p<0.05 reflected a violation of the assumption. Logistic regression was performed to assess the association between epidemiological variables and mutations in *pfcrt-*76 or *pfmdr1-*86, considering wild type as the reference category.

For both binary and cumulative logistic regression methods, the final most parsimonious multivariable model was selected based on biological plausibility, univariable analysis, and backwards elimination strategy (removal cutoff p>0.10) after assessing interaction and confounding. All possible two-way interaction terms with primary exposures of interest (drug use and survey year) and explanatory variables were examined using an overall likelihood ratio test. The likelihood ratio test was insignificant for interaction terms in all models using the α = 0.05 cutoff, so final statistical models did not contain any interaction terms. The final model contained the variables drug use (SP, antifolate, or CQ) and year of survey as the main predictors, and controlled for potential confounders including: age, parasite density, hemoglobin level, sex, and GIS distance to shore.

For all statistical tests, a two sided p<0.05 was considered to be statistically significant. Data and statistical analyses, including logistic regression, were performed using SAS software version 9.2 (SAS Institute Inc., Cary, NC, USA).

## Results

Of the 503 randomly selected parasite positive samples, 492 (97.8%) samples were successfully genotyped for all drug resistance SNPs, 250 (96.5%) for baseline and 242 (99.2%) for year 5 survey samples. Where genotyping failed for any of the markers, data was reported as missing.

### Characteristics of Study Participants

Participants at the year 5 survey were significantly older, lived farther from the lake shore and the nearest clinic, and had a higher hemoglobin level, and lower geometric mean parasite density compared to those at baseline. Significantly more SP but less CQ use was reported by participants in the year 5 survey than in baseline. Other characteristics including sex, report of fever, presence of gametocytes, elevation of compound, use of CTX, and use of any antimalarial did not differ significantly between year 5 and baseline survey samples (table 1).

**Table 1:** Characteristics of study participants at baseline and year 5 survey.

Category/Characteristics	Baseline (n = 250)	Year 5 Survey (n = 242)	p-value
Male sex (%)	126/250 (50.4)	121/242 (50.0)	0.93
Age, mean (SD), months	23.8 (15.0)	43.3 (17.6)	<0.0001*
Fever^a^ (%)	23/239 (9.6)	21/241 (8.7)	0.73
Hemoglobin, mean (SD), g/dL	7.9 (2.2)	10.2 (1.7)	<0.0001*
Parasite density^b^, geometric mean (95% CI), uL	2670 (2263–3156)	1339 (1048–1731)	<0.0001*
Gametocytes present (%)	43/250 (17.2)	56/242 (23.1)	0.1
Euclidean distance, mean (SD), meters			
To shore	7029 (2657)	7582 (2530)	0.018*
To nearest clinic	2255 (1017)	2534 (970)	0.0019*
Elevation of compound	1247 (52)	1251 (46)	0.33
Drug use^c^ (%)			
SP	4/246 (1.6)	17/236 (7.2)	0.0027*
Cotrimoxazole	13/247 (5.3)	20/242 (8.3)	0.19
CQ	46/247 (18.6)	25/236 (10.6)	0.013*
CQ or SP	50/250 (20.0)	42/242 (17.4)	0.45
Bednet usage^d^ (%)	–	223/242 (92.2)	–

**NOTE.** Data are proportion (%) of *P. falciparum* smear-positive participants with molecular data, unless otherwise indicated. SD, standard deviation; CI, confidence interval; SP, sulfadoxine-pyrimethamine; CQ, chloroquine.

a Body temperature greater than or equal to 37.5°C at or 48 hours before survey.

b Parasite density was log transformed prior to statistical analysis.

c Within two weeks prior to survey.

d Child slept under bednet >5 days in the past week, bednet usage in the study area was <5% prior to trial.

*P<0.05, statistically significant difference between baseline and year 5 survey based on chi-squared or two sample t-test (satterthwaite).

### Prevalence of SNP Mutations and SP and CQ Genotypes

**Figure 1:**
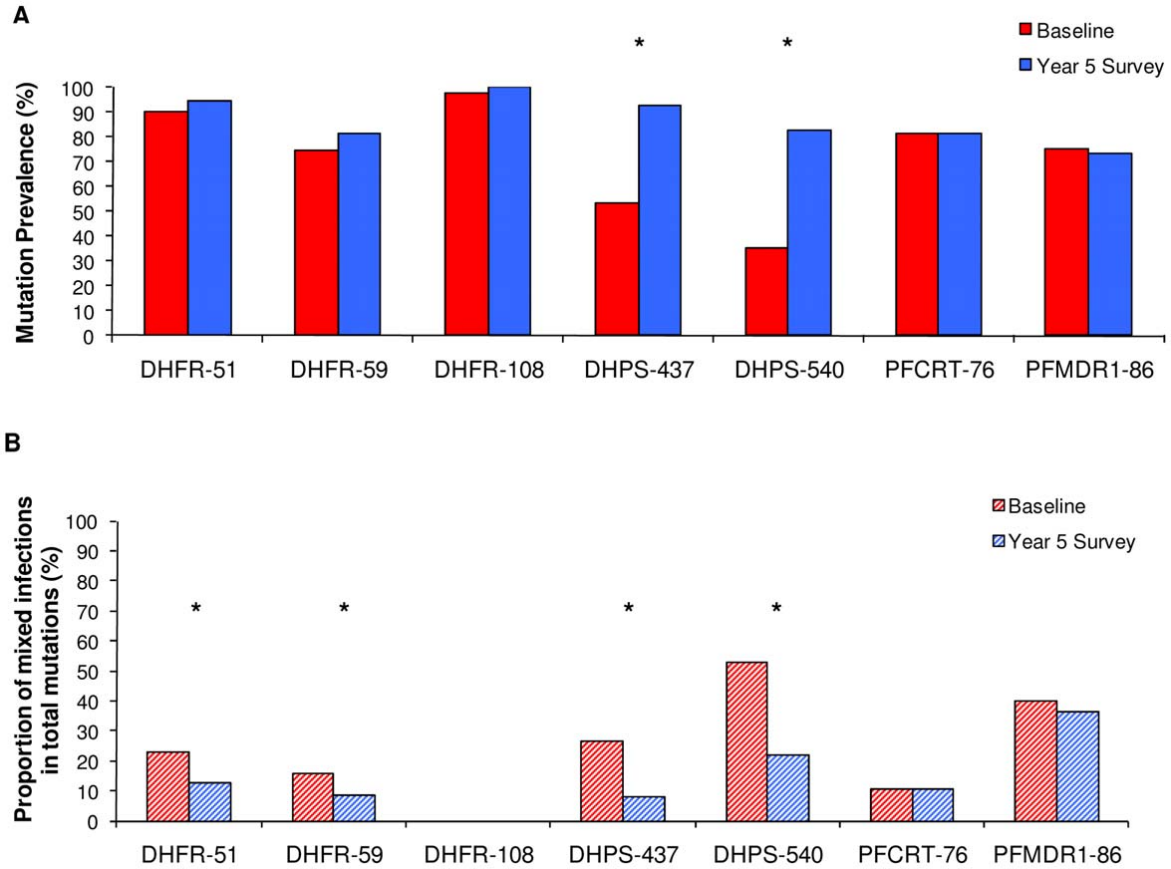
Comparison of mutation prevalence by SNP between baseline and year 5 survey. *A,* Overall prevalence of mutations ((pure mutations and mixed)/total samples). *B,* Proportion of mixed infections in total mutations (mixed/(pure mutations and mixed)). Statistical analysis performed using chi-squared test. * p<0.05, significant difference in prevalence between baseline and year 5 surveys.

The prevalence of mutations and proportion of mixed infections by SNP at baseline and the year 5 survey are summarized in figure 1. The prevalence of SNP mutations in *dhfr* and CQ-linked genes were high initially and remained unchanged at the year 5 survey, with non-significant increases from 90.4% to 94.6% for *dhfr*-51, 74.8% to 81.8% for *dhfr-59*, 97.6% to 100% for *dhfr-*108, 81.6% to 81.8% for *pfcrt-*76, and a non-significant decrease from 75.1% to 73.4% for *pfmdr1-*86. In contrast, a statistically significant increase (p<0.001) in the prevalence of mutations in both *dhps* codons was observed from baseline at 53.2% and 35.6% to the year 5 survey at 92.3% and 82.6% for *dhps-*437 and *dhps-*540, respectively (figure 1a). Of the total mutations (pure and mixed), the proportion of mixed infections detected at *dhfr*-51 (p = 0.0039), *dhfr*-59 (p = 0.036), *dhps*-437 (p<0.0001), and *dhps*-540 (p<0.0001) was significantly lower at the year 5 survey compared to baseline (figure 1b). There was no significant change in the proportion of mixed infections measured by *pfcrt-*76 and *pfmdr1-*86 SNPs and all samples were pure mutant type for *dhfr-*108 except for one mixed sample at the year 5 survey. No mutations in *dhfr*-164 were found at either survey.

**Figure 2:**
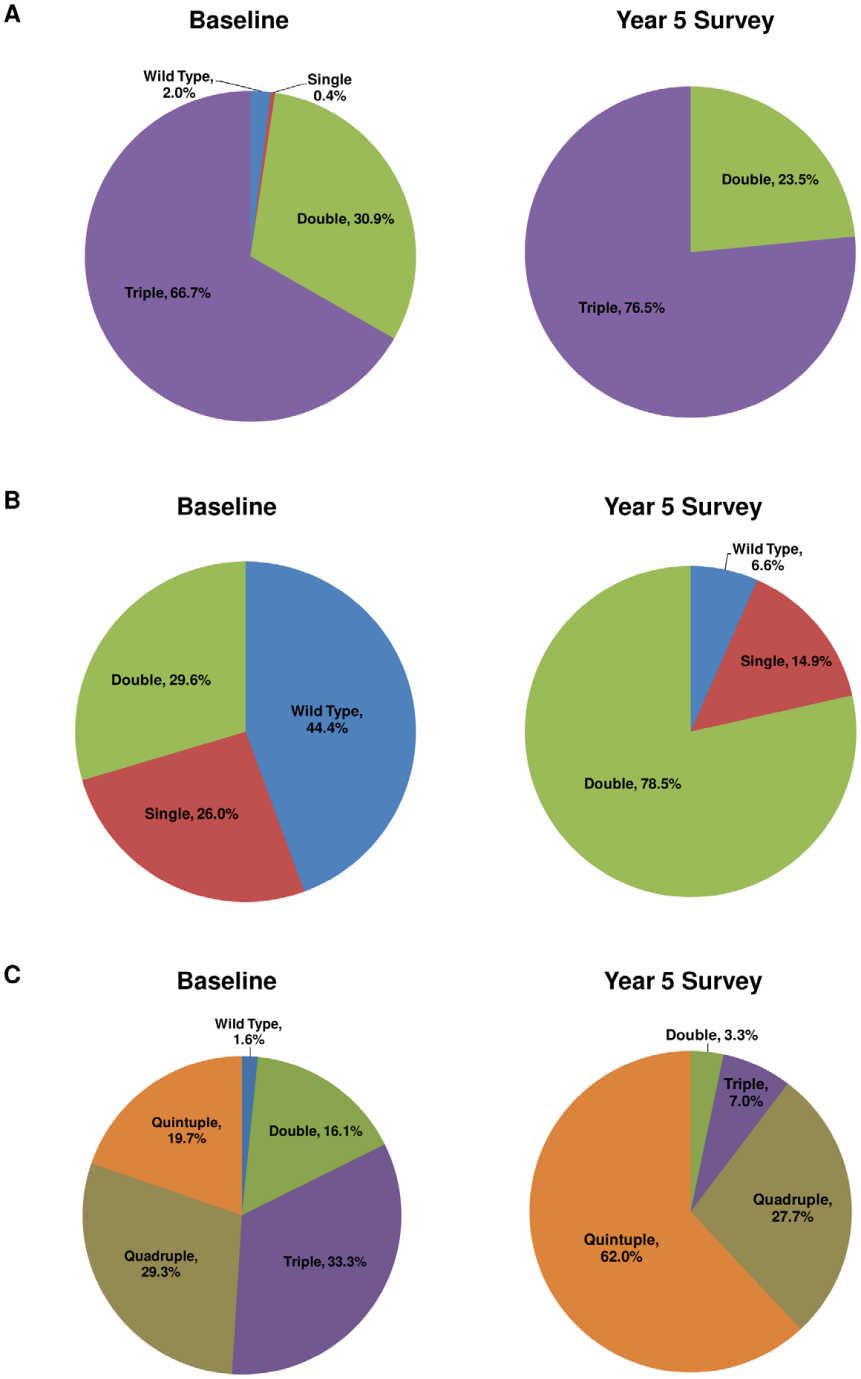
Prevalence of SP genotypes at baseline and year 5 survey. *A, dhfr* genotype based on mutations in *dhfr-*51,59,108. *B, dhps* genotype based on mutations in *dhps-*437,540. *C, dhfr/dhps* combined genotype based on *dhfr* and *dhps* genotypes. Statistical analysis performed using chi-squared test. The prevalence of *dhfr*, *dhps*, and *dhfr/dhps* combined genotypes were significantly different at baseline compared to the year 5 survey, p<0.05.

The overall prevalence of mutations in *dhfr* (p = 0.0075), *dhps* (p<0.0001), and *dhfr/dhps* combined (p<0.0001) genotypes was significantly different at baseline compared to the year 5 survey (figure 2). The *dhfr* triple mutant prevalence increased from 66.7% to 76.5%, while the *dhps* double mutant changed more dramatically, from 29.6% to 78.5%, from baseline to the year 5 survey (figure 2a, 2b). Quintuple mutations increased from 29.3% at baseline to 62.0% at the year 5 survey corresponding to a decrease in double and triple mutations (figure 2c).

### Haplotypes of Combined CQ and SP SNP Mutations

In order to evaluate the linkage between multiple mutations of 4 different genes, *crt*, *mdr1*, *dhfr* and *dhps* [28], [29], haplotypes of combined CQ and SP markers were constructed using single infection for all seven SNPs (table 2). The wild type haplotype, K_76_N_86_N_51_C_59_S_108_-A_437_K_540_, was not detected at either survey. Thirty-two distinct CQ-SP combined haplotypes were present at baseline, while only 23 were present at the year 5 survey. The distinct combined haplotypes detected at the pre- and post-intervention periods, respectively, comprised of 2 or 0 haplotypes in single mutants, 2 or 1 haplotypes in double mutants, 5 or 1 haplotypes in triple mutants, 5 shared and 2 different haplotypes in quadruple mutants, 10 or 8 haplotypes in quintuple mutants, 5 haplotypes in sextuple mutants, and 1 haplotype in septuple mutant isolates. Most importantly, both the sextuple haplotype,T_76_N_86_I_51_R_59_N_108_-G_437_E_540_ (containing *pfmdr1*-86 wild type), and septuple mutant haplotype, T_76_Y_86_I_51_R_59_N_108_-G_437_E_540_, increased dramatically from 1.08% and 5.4% at baseline to 20.72% and 27.9% at the year 5 survey, respectively (table 2). In addition, the haplotypes containing *pfcrt*-76 and *dhfr*-51, 59 and 108 mutations only, regardless of *pfmdr1*-86, T_76_N_86_I_51_R_59_N_108_-A_437_K_540_ and T_76_Y_86_I_51_R_59_N_108_-A_437_K_540_, significantly decreased from 9.68% and 13.98% to 1.8% respectively (table 2).

**Table 2:** Haplotypes for CQ and SP resistance markers combined at baseline and year 5 survey.

CQ and SP Haplotype^a^^,^ b	*crt76*	*mdr1*86	*dhfr* 51	*dhfr* 59	*dhfr* 108	*dhps*437	*dhps*540	Number of mutations	Baseline (n = 93) N (%)	Year 5 Survey (n = 111) N (%)
Wild Type	K aaa	N aat	N aat	C tgt	S agc	A gct	K aaa			
Mutant	T aca	Y tat	I att	R cgt	N aac	G ggt	E gaa			
	K	N	N	C	S	A	K	0	0 (0)	0 (0)
	K	**Y**	N	C	S	A	K	1	1 (1.08)	0 (0)
	**T**	N	N	C	S	A	K	1	1 (1.08)	0 (0)
	**T**	**Y**	N	C	S	A	K	2	2 (2.15)	0 (0)
	K	N	**I**	C	**N**	A	K	2	3 (3.23)	1 (0.90)
	K	**Y**	**I**	C	**N**	A	K	3	4 (4.30)	0 (0)
	**T**	N	**I**	C	**N**	A	K	3	8 (8.60)	1 (0.90)
	K	**Y**	N	**R**	**N**	A	K	3	1 (1.08)	0 (0)
	**T**	N	N	**R**	**N**	A	K	3	1 (1.08)	0 (0)
	K	N	**I**	**R**	**N**	A	K	3	1 (1.08)	0 (0)
	**T**	**Y**	**I**	C	**N**	A	K	4	9 (9.68)	3 (2.70)
	**T**	**Y**	N	**R**	**N**	A	K	4	3 (3.23)	1 (0.90)
	K	**Y**	**I**	**R**	**N**	A	K	4	2 (2.15)	1 (0.90)
	**T**	N	**I**	**R**	**N**	A	K	4	9 (9.68)	2 (1.80)
	**T**	N	**I**	C	**N**	**G**	K	4	1 (1.08)	1 (0.90)
	**T**	N	N	**R**	**N**	**G**	K	4	0 (0)	2 (1.80)
	K	**Y**	**I**	C	**N**	**G**	K	4	1 (1.08)	0 (0)
	K	N	**I**	**R**	**N**	**G**	K	4	1 (1.08)	0 (0)
	K	N	**I**	C	**N**	**G**	**E**	4	0 (0)	1 (0.90)
	**T**	**Y**	**I**	**R**	**N**	A	K	5	13 (13.98)	2 (1.80)
	**T**	**Y**	N	**R**	**N**	**G**	K	5	1 (1.08)	0 (0)
	**T**	**Y**	**I**	C	**N**	**G**	K	5	3 (3.23)	2 (1.80)
	K	**Y**	**I**	**R**	**N**	**G**	K	5	1 (1.08)	1 (0.90)
	**T**	N	**I**	**R**	**N**	**G**	K	5	2 (2.15)	1 (0.90)
	K	**Y**	**I**	C	**N**	**G**	**E**	5	1 (1.08)	3 (2.70)
	K	**Y**	N	**R**	**N**	**G**	**E**	5	1 (1.08)	0 (0)
	**T**	N	**I**	C	**N**	**G**	**E**	5	3 (3.23)	7 (6.31)
	**T**	N	N	**R**	**N**	**G**	**E**	5	1 (1.08)	4 (3.60)
	K	N	**I**	**R**	**N**	**G**	**E**	5	1 (1.08)	3 (2.70)
	**T**	**Y**	**I**	**R**	**N**	**G**	K	6	2 (2.15)	4 (3.60)
	**T**	**Y**	N	**R**	**N**	**G**	**E**	6	2 (2.15)	5 (4.50)
	**T**	**Y**	**I**	C	**N**	**G**	**E**	6	6 (6.45)	11 (9.91)
	K	**Y**	**I**	**R**	**N**	**G**	**E**	6	2 (2.15)	1 (0.90)
	**T**	N	**I**	**R**	**N**	**G**	**E**	6	1 (1.08)	23 (20.72)
	**T**	**Y**	**I**	**R**	**N**	**G**	**E**	7	5 (5.38)	31 (27.93)

**NOTE.** CQ, chloroquine; SP, sulfadoxine-pyrimethamine.

a The construction of the CQ-SP haplotype included *pfcrt-76*, *pfmdr1 -86*, *dhfr-51,59,108*, and *dhps-437,540*. All samples are single infection for all seven SNPs.

b Wild type amino acids are shown in plain font, while mutated amino acids are depicted in bolded font.

### Association between Number of Mutations in SP and CQ Genotypes and Drug Use or Year of Survey

The final statistical model for the association analysis was selected based on biologic plausibility, results from univariable analysis, and backwards elimination strategy. The final model contained the variables drug use (SP, antifolate, or CQ) and year of survey as the main predictors, and controlled for age, parasite density, hemoglobin level, sex, and GIS distance to shore as potential confounding factors.

The relationship between drug use and drug resistance genotypes was studied by examining the effect of antifolate, SP, and CQ use on mutations in their corresponding molecular markers. Antifolate use was significantly associated with more mutations in *dhps* genotype (adjusted odds ratio [OR], 2.3 [95% confidence interval {CI}, 1.1–4.7]) and, in the univariable model only, more mutations in *dhfr/dhps* combined genotype (unadjusted OR, 2.2 [95% CI, 1.2–3.8]) (table 3). Among participants reporting SP use alone, the unadjusted odds for having more *dhps* mutations (unadjusted OR, 5.6 [95% CI, 1.6–19.4]) and more *dhfr/dhps* combined mutations (unadjusted OR, 3.0 [95% CI, 1.2–7.3]) were significantly higher than for those who reported no SP use (table 3). These associations did not remain significant after adjusting for sex, age, hemoglobin level, parasite density, and GIS distance to shore as well as year of survey. No significant associations between CQ use and mutations in *pfcrt*-76 or *pfmdr1*-86 were observed in either univariable or multivariable analysis (table 4).

**Table 3:** Univariable and multivariable analyses of the association between specific predictors and mutations in *dhfr*, *dhps*, and *dhfr/dhps* combined.

	*dhfr* Triple Mutant^a^		*dhps* Mutations^b^		*dhfr/dhps* Mutations^c^	
	OR (95% CI)		OR (95% CI)		OR (95% CI)	
Predictor	Unadjusted	Adjusted^d^	Unadjusted	Adjusted^e^	Unadjusted	Adjusted^e^
Antifolate use						
SP and/or CTX^f^	1.1 (0.6, 2.1)	0.9 (0.5, 1.8)	3.2 (1.6, 6.2)*	2.3 (1.1, 4.7)*	2.2 (1.2, 3.8)*	1.6 (0.9, 2.9)
SP only^g^	1.0 (0.4, 2.7)	0.8 (0.3, 2.1)	5.6 (1.6, 19.4)*	3.4 (0.9, 12.8)	3.0 (1.2, 7.3)*	1.9 (0.7,4.8)
Year of Survey^h^	1.6 (1.1, 2.4)*	1.6 (1.0, 2.6)	9.2 (6.2, 13.7)*	10.3 (6.3, 17.0)*	7.5 (5.2, 10.8)*	8.7 (5.5, 13.9)*

**NOTE.** CI, confidence interval; OR, odds ratio; SP, sulfadoxine-pyrimethamine; CTX, cotrimoxazole.

a *dhfr* triple mutant was analyzed by grouping wild type, single and double genotypes as the reference category.

b *dhps* mutations were analyzed as 3 genotype categories: (1) wild type, (2) single, and (3) double.

c *dhfr* and *dhps* combined mutations were analyzed as 3 genotype categories: (1) wild type, single, double, and triple, (2) quadruple, and (3) quintuple.

d Derived from multivariable logistic regression, controlling for age, sex, parasite density, hemoglobin level, and GIS distance to shore.

e Derived from multivariable cumulative logistic regression, which models the probability of more mutations compared to fewer, controlling for age, sex, parasite density, hemoglobin level, and GIS distance to shore.

f Model containing SP and/or CTX use as a predictor.

g Model containing SP use only as a predictor.

h Year 5 survey versus baseline.

*Statistically significant, p<0.05.

**Table 4:** Univariable and multivariable analyses of the association between specific predictors and mutations in CQ-linked drug resistance genes.

	*pfcrt*-76 Mutant^a^		*pfmdr1*-86 Mutant^a^	
	OR (95% CI)		OR (95% CI)	
Predictor	Unadjusted	Adjusted^b^	Unadjusted	Adjusted^b^
CQ use	1.0 (0.5, 1.9)	1.0 (0.5,1.9)	1.5 (0.8, 2.7)	1.5 (0.8, 2.8)
Year of Survey^c^	1.0 (0.6, 1.6)	1.1 (0.6, 2.0)	0.9 (0.6, 1.4)	0.8 (0.5, 1.3)

**NOTE.** CI, confidence interval; OR, odds ratio; CQ, chloroquine.

a *pfcrt*-76 and *pfmdr1*-86 mutants were analyzed using wild type as the reference group.

b Derived from multivariable logistic regression, controlling for age, sex, parasite density, hemoglobin level, and GIS distance to shore.

c Year 5 survey versus baseline.

*Statistically significant, p<0.05.

The association between the year of survey and SP and CQ genotypes was also explored. The unadjusted odds of having more mutations in *dhfr*, *dhps*, and *dhfr/dhps* combined genotypes at the year 5 survey were 1.6 ([95% CI, 1.1–2.4]), 9.2 ([95% CI, 6.2–13.7]), and 7.5 ([95% CI, 5.2–10.8]) times the odds of having more mutations at baseline, respectively (table 3). In multivariable analysis, the association remained significant for *dhps* genotype (adjusted OR, 10.3 [95% CI, 6.3–17.0]) and *dhfr/dhps* combined genotype (adjusted OR, 8.7 [95% CI, 5.5–13.9]) (table 3) after adjusting for antifolate drug use and other potential confounders. The year of survey was not associated with the *pfcrt*-76 or *pfmdr1*-86 mutant in either univariable or multivariable analysis (table 4).

## Discussion

With the scale-up of ITNs as a malaria control strategy [7], assessing the impact of the transmission-reducing intervention on the spread of drug resistance has become a particularly important concern. The current understanding of the relationship between transmission intensity and the spread of drug resistance relies on the effect of host, parasite, and vector factors [3], [12]. In this study, the effect of sustained transmission reduction by ITNs on the spread of drug resistance during a shift in national drug policy from CQ to SP was investigated. The prevalence of gene mutations associated with drug resistance to SP and CQ between baseline and five years post-ITN introduction was compared and explored further to understand how some variables may shape the dynamics of drug resistance.

The accumulation of mutations in the *dhfr* and *dhps* genes in response to drug pressure is stepwise and occurs first in the *dhfr* gene. In this study, there was a high prevalence of the *dhfr* triple mutant parasite (67%) but a relatively lower prevalence of the *dhps* double mutant (30%) at baseline. Although SP use in the study area was limited due to poor availability before the national drug policy change in 1998 [23], the high prevalence of mutations in *dhfr* gene at the baseline (1996) could be attributed to both the on-going use of CTX, which is also an inhibitor of the *dhfr* and *dhps* enzymes [31] and the possible gene flow due to human migration from the surrounding bay area, where SP use was relatively common [32] , to this study area. At the year 5 survey, there was a significant increase in the prevalence of the *dhps* double mutant (from 30% to 79%) and *dhfr/dhps* quintuple mutations (from 20% to 62%), but a less dramatic increase in *dhfr triple* mutant (from 67% to 77%) (figure 2). Further analysis showed that SP use alone and antifolate use (SP and/or CTX) were associated with more mutations in *dhps* genotype and *dhfr/dhps* combined genotype, although only the association of antifolate use with more mutations in *dhps* genotype remained statistically significant after controlling for multiple confounding factors (table 3). Taken together, it is clear that increased antifolate drug use, most likely resulting from drug policy change, played a predominant role in the selection of SP resistant parasites, leading to the high prevalence of *dhps* double and *dhfr/dhps* quintuple mutations during the period of ITN intervention. The results from the children in this study are consistent with the increased prevalence of *dhfr* and *dhps* mutations observed in all age groups in Kisumu, Kenya as well as in Tanzania after drug policy changed to SP or CTX [33], [34], [35].

The current study further showed a strong and significant association between the year 5 survey and more mutations in *dhps* and *dhfr/dhps* combined genotypes (table 3). This association remained distinct after adjusting for antifolate drug use and other potential confounders, suggesting that the differences in other factors between the two survey time points may have regulated the high prevalence of SP mutations observed at the year 5 survey. This speculation could rely on the following plausible explanations of a few proxy factors.

Reduction in transmission intensity presumably lowers the multiplicity of clonal infections [3]. However, a previous investigation by our group using neutral microsatellite markers in a subset of samples used in the present study showed an unchanged, high overall clonal multiplicity despite the reduction in EIR five years post-ITN intervention [30]. The counter-intuitive result from the previous study was surprising and suggested a strong resilience of the malaria parasite in response to dramatic transmission reduction after five years of sustained ITN use. Although the unchanged high level of overall clonal multiplicity alone has no effect on the spread of drug resistance for the monogenic-based drug resistant genes, such as SP, the rate of drug resistance could increase in the presence of intrahost competition between co-infecting parasite clones (intrahost dynamics) based on generalized immunity model [36]. In the current study, a significant decrease in the proportion of mixed infections out of total mutations for *dhfr-*51, 59 and *dhps-*437, 540 SNPs were observed at the year 5 survey compared to baseline (figure 1B). This result suggests that intrahost removal of SP drug wild/sensitive parasite clones is present at the year 5 survey, thus purifying and expanding drug resistant clones at population level. However, it is unclear the exact interplay between altered host immunity due to the transmission reduction by ITN use (not measured here) and increased antifolate use in the removal of SP drug sensitive parasites [36].

At baseline, the prevalence of *pfcrt-*76 and *pfmdr1-*86 mutations was high at 82% and 75%, respectively, which presumably resulted from the progression of CQ drug pressure. Despite decreased CQ drug use due to the change in first line antimalarial treatment to SP at second year of ITNs trial period and decreased malaria case treatment due to the transmission reduction by use of ITNs [23], the high prevalence of CQ gene mutations remained unchanged between baseline and the year 5 survey (82% and 73%, respectively). This result could have several explanations. First, the time frame evaluated in this study may not be sufficient to observe considerable decreases in mutation prevalence of CQ molecular markers at such high levels; therefore, long term monitoring is necessary to detect changes in the prevalence of mutations. A study conducted in Malawi showed that *pfcrt* mutant *g*enotype significantly declined after cessation of CQ use for eight years [37], [38]. Second, CQ was not completely withdrawn from Kenya after the drug policy shift to SP in 1998 as shown in table 1, hence, CQ drug pressure presumably was still present. In the year 5 survey that occurred 3 years after the national drug policy change to SP, 10.6% of participants reported using CQ (table 1), suggesting that CQ was still available and used in this study area after the policy shift to SP. Third, the unchanged overall high clonal multiplicity measured by neutral microsatellite markers as mentioned before [30] and the absence of intrahost competition measured by the proportion of mixed infection using CQ markers in the current study suggest that transmission reduction by the use of ITNs does not affect the prevalence of CQ mutations. This explanation corroborates with the theoretical models described earlier [3], [12].

In this study, the haplotypes constructed from CQ and SP resistance gene SNPs located on different chromosomes were further analyzed to gain insight into genetic linkage of the multiple mutations and transmission dynamics of drug resistant parasites. Many of the haplotypes detected in this study have been reported previously in Africa [28], [29]. Although the most dramatic increase was noted in the septuple mutant, T_76_Y_86_I_51_R_59_N_108_G_437_E_540_, from baseline to the year 5 survey, there was also a substantial increase in isolates containing the *pfmdr1-86* sensitive sextuple mutant, T_76_N_86_I_51_R_59_N_108_G_437_E_540_. Additionally, there was a significant decrease in the haplotypes containing *dhfr*-51, 59 and 108 mutations and sensitive *dhps*-437 and 540 (T_76_N_86_I_51_R_59_N_108_A_437_K_540_ and T_76_Y_86_I_51_R_59_N_108_A_437_K_540_) from baseline to the year 5 survey. These results revealed a clear selection of *dhps* mutations (A_437_K_540_ to G_437_E_540_) due to increased SP pressure after policy implementation. The result, strong genetic linkage between mutations for CQ, particularly *pfcrt*76, and the accumulation at *dhfr* and *dhps* mutations for SP as expected, also suggests that separate mutations conferring resistance to unrelated drugs could be coordinated beyond the chromosomal level [28], [29] although molecular pathways involved in the process are still unknown. The strong linkage between CQ and SP mutations may also offer a partial explanation to the unchanged high prevalence of *pfcrt*-76 mutation (80%) in this study, in which fast accumulation of *dhfr* and *dhps* mutations driven by increased SP pressure may play a role in preventing the recovery of parasites with wild type *pfcrt* (20%). However, this speculation requires further investigation. Importantly, the higher prevalence of parasites with CQ and SP combined sextuple and septuple mutant haplotypes that circulated in this study area in the year 5 survey calls for the continuation of monitoring both CQ and SP molecular markers in this area.

There were a few limitations of the current study. First, this study compared two independent populations from the same study site at two different points in time; hence the results of drug resistant molecular markers might be influenced by unmeasured factors that changed in the study site over the 5 year study period in addition to the factors investigated. To the best of our knowledge, other than the ITNs trial and the change in antimalarial drug policy no other malaria interventions and associated epidemiological impacts occurred in the study area during the study period. Despite this, the absence of a nearby comparison area without community ITN use at the year 5 survey [18] limited comparisons to quantify the contribution of transmission reduction by ITN use on the spread of drug resistance in this study. Second, it is not clear whether drug use as measured in the current study reflects the proportion of therapeutic drug use that is mainly influenced by acquired immunity and/or the level of community drug use that is regulated by infection risk. Consequently, it was not possible to completely to dissect the role of drug pressure in these results. It would be ideal to conduct well-controlled studies in areas with dramatically different levels of ITN coverage/usage during the same time period with no drug policy change, since comparing sites where ITNs have not been distributed to date may not be feasible and ethical. Such studies would help to assess the net effect of transmission reduction by ITNs on the spread of antimalarial drug resistance.

The findings from this study differ with the results from the studies conducted in Tanzania and Burkina Faso which also assessed the short or long term effects of ITNs or ITCs on prevalence of gene mutations linked to SP and CQ, respectively [9], [11]. The discrepancy in the results among the studies could be due to differences in the study design, level of transmission reduction, stage of existing drug resistance, change in drug policy during the study, and other potential confounders. In the Tanzania study, a reduction in the prevalence of *dhfr* triple mutation was observed two years after ITN introduction and during the two year study period SP was first line drug treatment for malaria. The study conducted in Burkina Faso, where CQ remained first line treatment during study period, reported no change in the prevalence of molecular markers linked to CQ and SP after seven years of ITC intervention. The present study showed that increased antifolate drug use likely led to the high prevalence of SP mutations five years post-ITN intervention and reduced transmission did not change the existing high prevalence of CQ mutations. In addition, the difference in the degree of local gene flow resulting from human movement between intervention and non-intervention areas could be another factor for the inconsistent results among the different studies. Although the previous short-term study concluded that transmission reducing interventions such as ITNs may help restore the susceptibility of SP [9], there is no evidence from the current study that sustained transmission reduction by ITNs reduces the prevalence of drug resistance genes associated with SP and CQ.
